# Effect of Depletion of Developmentally Regulated GTP Binding Protein on Osteoblastic Differentiation and Bone Microarchitecture

**DOI:** 10.1111/jcmm.70895

**Published:** 2025-10-29

**Authors:** Yuan‐Zhe Jin, Minjoon Cho, Jae Hyup Lee

**Affiliations:** ^1^ Department of Orthopedic Surgery, College of Medicine Seoul National University Seoul Korea; ^2^ Department of Orthopedic Surgery The First Hospital of Jilin University Changchun China; ^3^ Department of Orthopedic Surgery, SMG‐SNU Boramae Medical Center Seoul Korea; ^4^ Institute of Engineering Research Seoul National University Seoul Republic of Korea

**Keywords:** bone formation marker, bone mineral density, DRG2, knockout mouse, osteoblast

## Abstract

Previous studies have demonstrated that overexpression of DRG2 enhances the osteoclastic activity. However, its impact on osteoblastic differentiation remains unexplored. This study investigates the relationship between DRG2 and osteoblastic activity through in vitro and in vivo studies. DRG2 expression was inhibited in mouse MC3T3‐E1 cells, and its effects on the expression of bone formation markers and osteoblast‐related transcription factors were evaluated. The capacity for osteoblastic differentiation was assessed in mouse preosteoblasts following DRG2 gene knockdown using a short hairpin RNA (shRNA) plasmid. Female C57BL/6N strain DRG2 knockout (KO) mice were generated, and bone phenotypes of the vertebrae and femurs were analysed. Additionally, osteoblastic differentiation capacity was evaluated in bone marrow mesenchymal stem cells (BM‐MSCs) isolated from these mice under physiological and ovariectomized conditions. Inhibition of DRG2 in MC3T3‐E1 cells resulted in upregulation of bone formation markers and osteoblast transcription factors. BM‐MSCs from DRG2 KO mice exhibited significantly higher osteoblastogenesis than wild‐type (WT) mice. DRG2 KO mice demonstrated significantly increased percent bone volume and bone mineral density (BMD) in the vertebra and femur compared to WT mice under physiological and ovariectomized conditions. The inhibition of DRG2 promotes osteoblastic differentiation and is associated with increased BMD, suggesting its potential role in enhancing bone formation.

## 1 Introduction

Understanding genes associated with diseases and elucidating molecular mechanisms are pivotal in managing diseases within contemporary medicine. Pertaining to osteoporosis, understanding of the Receptor Activator of Nuclear Factor‐κB Ligand (RANKL) signalling pathway and the role of sclerostin protein have enhanced comprehension of the disease and facilitated the development of therapeutic agents such as denosumab and romosozumab [1, 2, 3]. Treatments employing these monoclonal antibodies offer a novel approach for numerous physicians, enabling more efficacious interventions for osteoporosis compared to the previously limited drug options [1, 2, 4, 5, 6], such as bisphosphonates [7, 8].

DRG 2 is a highly evolutionarily conserved protein and one of the paralogs belonging to the developmentally regulated GTP‐binding protein (DRG) subfamily, playing crucial roles in the modulation of cell proliferation, translation, and microtubule dynamics [9, 10, 11]. In addition to its known roles in cell cycle regulation [10, 12], immune function [13, 14], and mitochondrial function [15], emerging evidence suggests its involvement in cellular senescence [16] and protein trafficking [17]. However, its role in bone metabolism remains largely unexplored.

From a bone‐related perspective, a few studies suggest that the overexpression of DRG2 may impact bone resorption by influencing osteoclastic activity.

Ke et al. reported that DRG2 overexpression induces bone loss by increasing osteoclast activity [18]. However, in contrast, Lim et al. previously presented reduced ossification in the skull of DRG2 knockout (KO) mice, suggesting a potential opposing role of DRG2 in bone metabolism [18, 19]. Despite these findings, no further investigations have examined the effect of DRG2 on osteoblastic differentiation.

Since bone homeostasis relies on a delicate balance between bone resorption and bone formation, it is imperative to investigate osteoblast differentiation to elucidate DRG2's potential role in bone metabolism. Furthermore, considering that DRG2 modulates key cellular processes such as cell cycle regulation, mitochondrial function, and immune modulation, its involvement in osteoporosis—especially postmenopausal osteoporosis, a representative manifestation of the disease—remains an open question.

This study aims to investigate whether DRG 2 modulates osteoblastic differentiation and bone mineral density (BMD) through key signaling pathways, including BMP, Smad, and MAPK, which are already well established in bone biology. By understanding its role, we may uncover new mechanistic insights into bone homeostasis and potential therapeutic targets for osteoporosis.

## 2 Materials and Methods

To investigate the role of DRG2 in vitro, MC3T3‐E1 cells were transfected with DRG2‐specific shRNA. Control and transfected groups were cultured in growth medium (GM) and osteogenic medium (OM), respectively, to assess their influence on osteoblastic differentiation. Subsequently, ALP (alkaline phosphatase) assays and calcium assays were performed at day 7, 14, and 21. ALP and calcium staining were conducted at day 3, 7, 14, and 21 for comparison. Bone metabolism was assessed by examining bone formation markers BSP (bone sialoprotein), OPN (osteopontin), OCN (osteocalcin), ALP, and COL1 at day 3, 7, and 14. To determine the association between DRG2 knockout and osteoblastic differentiation, the expression levels of transcription factors related to osteoblastic differentiation were evaluated in vitro. Additionally, the expression levels of various genes related to canonical and non‐canonical BMP signalling pathways were examined to confirm their association with osteoblastic differentiation via real‐time PCR. Detailed protocols for shRNA transfection of MC3T3‐E1 cells, osteoblastic differentiation, ALP assays, calcium assays, PCR, and RT‐PCR are provided in the Supporting Information.

DRG2 knockout mice were generated using the CRISPR/Cas9 technique on the C57BL/6N strain at Korea Research Institute of Bioscience and Biotechnology (KRIBB) to further investigate the role of DRG2. All procedures involving the use of animals were officially approved. Mice were maintained for more than nine generations by mating between heterozygous mice. At 8 weeks of age, genotyping confirmed the DRG2 knockout status, and serum levels of procollagen type 1 N‐terminal propeptide (P1NP) and C‐terminal telopeptide of type 1 collagen (CTX) were measured and compared between wild‐type and knockout mice using ELISA. Bone marrow MSCs were extracted from the femur and tibia of wild‐type and DRG2 knockout mice. ALP assay and calcium assay were performed at days 3, 7, and 14, while ALP staining and calcium staining were conducted at days 3, 7, 14, and 21. Phenotypic analysis of bone‐related traits in KO mice was conducted by performing ovariectomy to induce osteoporotic conditions, in addition to the wild‐type and KO groups. Micro‐CT imaging of vertebrae and femurs was performed for each group to measure BMD and bone parameters.

### 2.1 Fabrication of DRG2 Knockout Mice

For mutant lineage establishment, the founder mouse was crossed with C57BL/6N mice to maintain a pure C57BL/6 background. Three mutant founders were fabricated. The #2 founder (49 bp deletion in exon 3) and #7 founder (31 bp deletion in exon 3) were sterile. Therefore, the #4 founder with a 48 bp deletion in exon 3 was crossed with C57BL/6N mice in this study (Figure S1). We have adhered to the ARRIVE guidelines and have included the ARRIVE checklist.

### 2.2 Genomic Typing and Gender Determination

For genomic typing, 2–3 mm tail tissue was dissected under gaseous anaesthesia. Tail tissue was lysed using a genomic DNA extraction kit (iNtRON Biotechnology Inc., Korea). PCR amplification was conducted with Maxime PCR Premix (i‐Star Taq) (iNtRON Biotechnology Inc., Korea). Sequences of primers were listed in Table S1.

### 2.3 Primary Culture of Bone Marrow MSC

The mice were anaesthetised with a zoletil/xylazine (20 mg/kg and 10 mg/kg, respectively) mixture and euthanized via CO_2_ inhalation. The bone marrow inside the tibia and femur was flushed out with a basic culturing medium (DMEM low glucose, WELGENE, Cat. LM 001‐12; 10% FBS and 1% antibiotic antimycotic) and was collected in a 10 mL tube. For culturing BMSCs, the flushed‐out cells were cultured in 10 cm dishes, and the medium was changed after 2 h.

### 2.4 Serum P1NP and CTX Measurement

Eight weeks of female mice were anaesthetised with a zoletil/xylazine (20 and 10 mg/kg, respectively) mixture. After general anaesthesia, the blood was collected into a serum separation tube.

Serum P1NP and CTX levels were measured with specific ELISA kits for mouse P1NP (MyBioSource, #X03147743) and CTX (MyBioSource, #32289442). Sensitivity was 0.6 ng/mL for CTX and 1.17 pg/mL for P1NP. Intra‐ and inter‐assay variation coefficients were ≤ 8.0% and ≤ 12% for the CTX kit and < 8.0% and < 10% for the P1NP kit, respectively.

### 2.5 Ovariectomy of Mice

Eight‐week‐old female mice were anaesthetised with Zoletil and xylazine. In the ovariectomy (OVX) group, the ovaries were surgically removed, while in the sham group, the ovaries were left intact. All animal procedures were IACUC‐approved (SSBMC‐IACUC No. 2017‐0007). Sixteen weeks post‐surgery, the mice were euthanized, and their spines and femurs were harvested for analysis. The mice were randomly assigned to sham or OVX groups using Excel (Microsoft).

### 2.6 Micro‐CT Evaluation

The microarchitectures of the lumbar vertebra were compared between the WT‐sham group (*N* = 25), KO‐sham group (*N* = 30), WT‐OVX group (*N* = 28), and KO‐OVX group (*N* = 30) via Micro‐CT. The microarchitectures of the femur neck were also compared between the WT‐sham group (*N* = 11), KO‐sham group (*N* = 12), WT‐OVX group (*N* = 12), and KO‐OVX group (*N* = 12).

Tomography projections of samples were acquired using a Skyscan 1172 micro‐CT scanner (Bruker, Belgium). Trabecular bone thickness (Tb.Th) and percentage bone volume (BV/TV) were calculated within 8‐mm diameter regions of interest (ROI), generated based on the defect site. In spine samples, the ROI was located in the caudal region of a vertebra (Figure 8c). In the femur neck, the ratio of outer and inner cortical bone perimeter (Outer.Pm/Inner.Pm ratio), cortical bone thickness (C.th), and BMD of the femur neck and BMD of proximal femur were analyzed (ROI shown in Figure 8c).

### 2.7 Statistics

For in vivo data, each “n” value corresponds to a single sample. For in vitro data, each “n” value corresponds to an independent well. All data are presented as mean ± standard deviation. The analysis was performed with one‐way ANOVA, followed by Bonferroni's multiple comparisons test.

All analyses were performed with SPSS (IBM) and GraphPad Prism (Version 8). The data were presented as mean ± SD, and the error bars represented the standard deviation.

## 3 Results

### 3.1 Knockdown the Expression Level of DRG2 Increased Osteoblastic Differentiation of MC3T3‐E1 Cells

**FIGURE 1:**
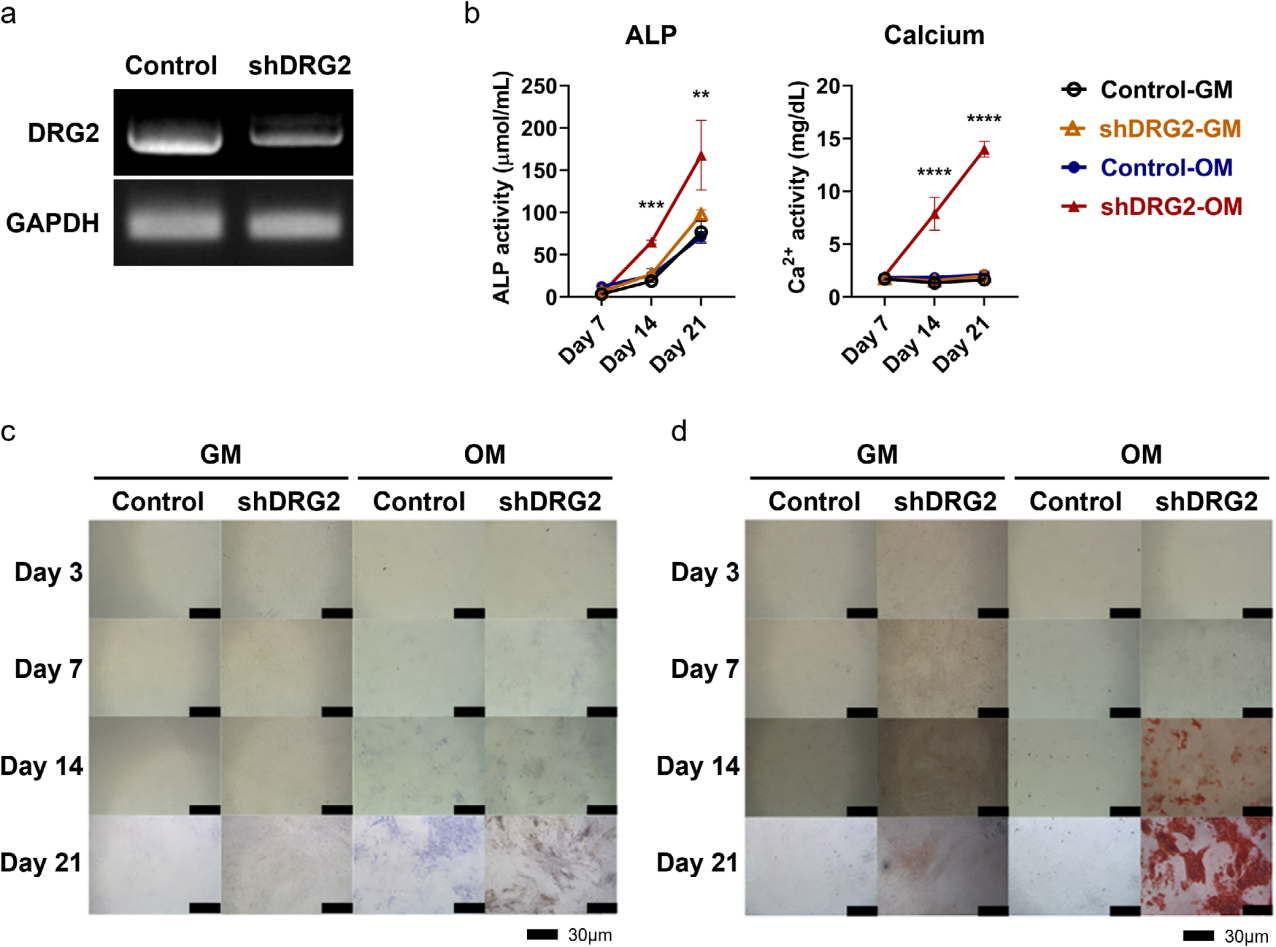
Results of DRG2 expression, ALP assay, Calcium assay of shRNA‐treated MC3T3‐E1 cells, and the result of ALP staining in different mediums. (a) DRG2 gene expression was diminished in MC3T3‐E1 cells. (b) ALP, Ca assay results showed the shDRG2‐OM groups showed significantly higher level than the GM groups on day 14 and 21. (c) The result of ALP staining. shRNA‐OM group showed denser ALP staining than the other groups from the day 14. (d) Result of Calcium staining. shRNA‐OM group showed denser calcium staining than the other groups from day 14. GM, growth medium; OM, osteoinductive medium. *p* < 0.05, **p < 0.01, ***p < 0.001, ****p < 0.0001.

The expression of DRG2 was significantly lower in the shRNA‐treated group than in the control group (Figure 1a). The shRNA‐OM group had significantly higher ALP activity on day 14 and 21 than the control‐OM group (day 14, *p* < 0.001; day 21, *p* < 0.01). Additionally, the shRNA‐basic group showed higher ALP activity on day 14 than the control‐basic group. In the calcium assay, the shRNA‐OM group showed significantly higher calcium concentration than the control‐OM group on day 14 and 21 (day 14, *p* < 0.0001; day 21, *p* < 0.0001) (Figure 1b). In the ALP staining, shRNA‐treated E1 cells showed denser ALP staining than control cells after induction with OM on day 7, 14, and 21 (Figure 1c). Alizarin red staining results also showed more evident mineral deposition compared to control E1 cells after treatment with OM on day 14 and 21 (Figure 1d).

**FIGURE 2:**
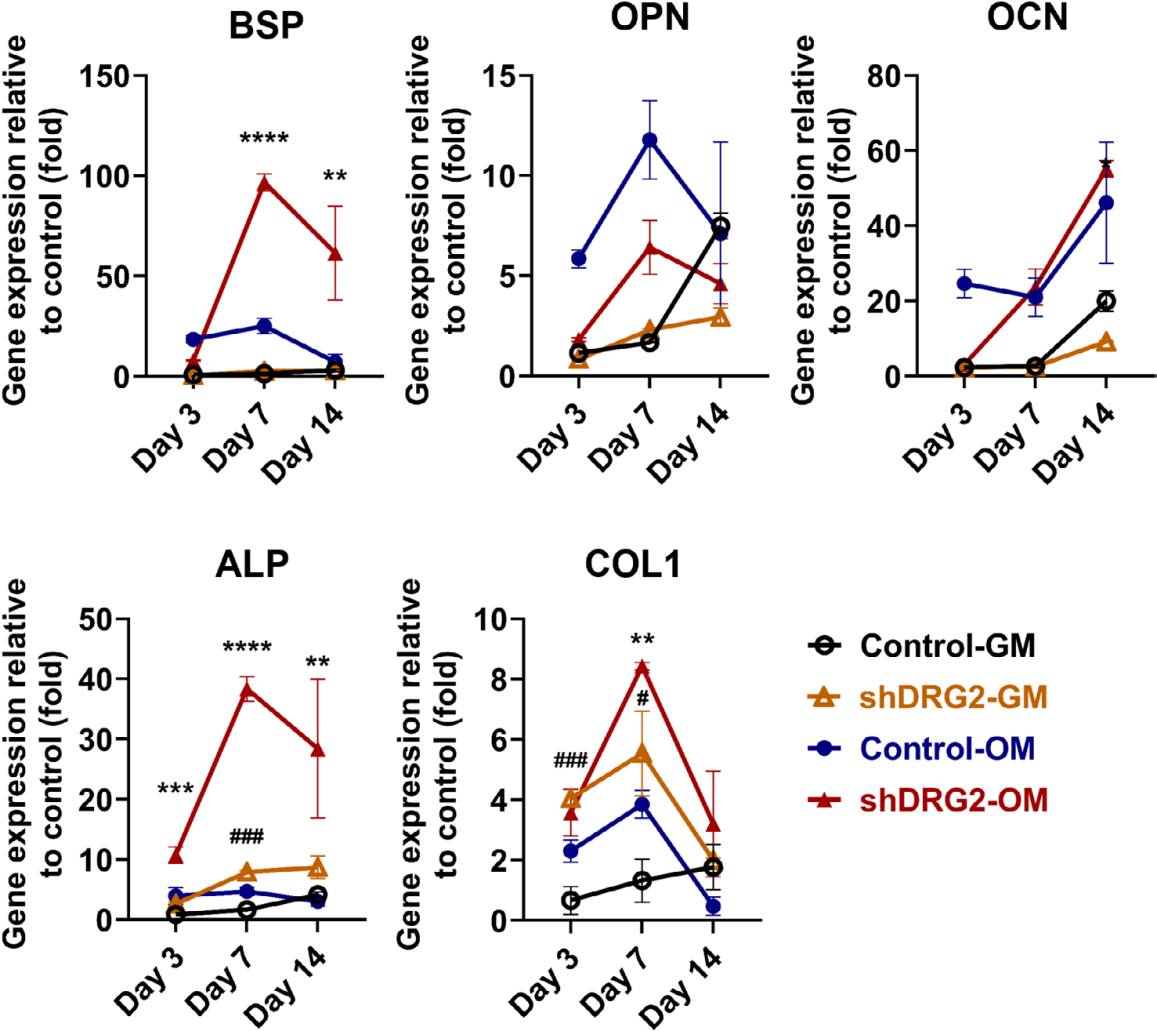
Bone formation markers in MC3T3‐E1 cells. The transcription level of BSP, ALP, and COL1 were significantly higher in the shDRG2‐OM group. BSP, bone sialoprotein, ALP, alkaline phosphatase: COL1, collagen type 1; GM, growth medium; OCN, osteocalcin; OM, osteoinductive medium; OPN, osteopontin. *p < 0.05, **p < 0.01, ***p < 0.001, ****p < 0.0001 versus control; ###*p* < 0.001 versus DRG2 knockdown group.

The expression level of BSP was higher in the control‐OM group than the shRNA‐OM group on day 3 but became significantly higher in the shRNA‐OM group on day 7 and 14 (day 7, *p* < 0.0001; day 14, *p* < 0.001). The expression of OPN was found to be higher in control‐OM on day 3, 7, and 14. The expression level of OCN was higher in the control‐OM group than the shRNA‐OM group on day 3 but was higher in the shDRG2‐OM group. The expression level of Alp was significantly higher in the shRNA‐OM group on days 3, 7, and 14 (day 3, *p* < 0.001; day 7, *p* < 0.0001; day 14, *p* < 0.001). The expression level of Alp was significantly higher in the shDRG2‐GM group compared with the control‐GM group on day 7 (*p* < 0.001). The expression level of Col1 was significantly higher in the shRNA‐OM group compared with the control‐OM group on day 7 (*p* < 0.01). The expression level of Col1 was significantly higher in the shDRG2‐GM group than the control‐GM group on days 3 and 7 (day 3, *p* < 0.001; day 7, *p* < 0.05) (Figure 2).

**FIGURE 3:**
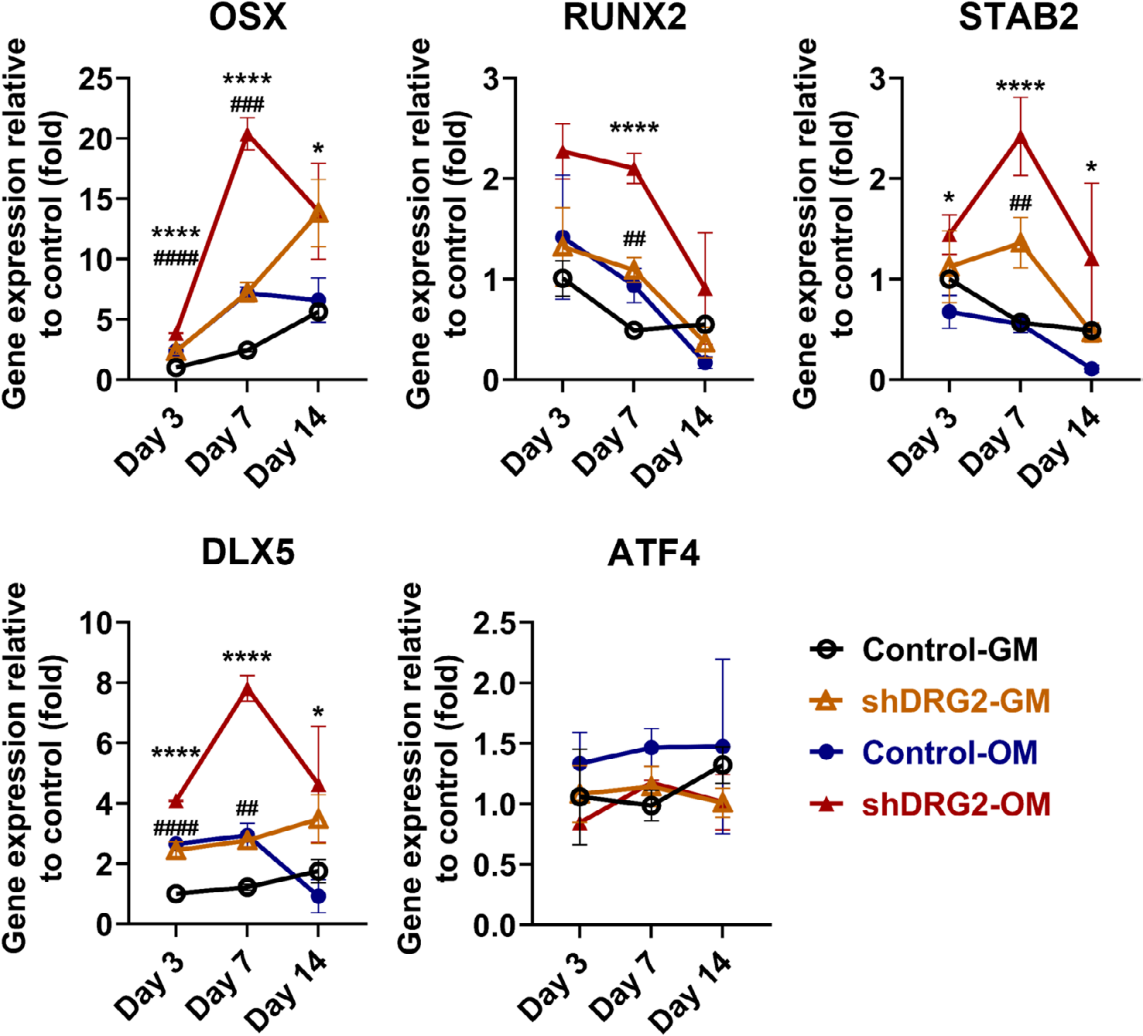
Expression levels of osteoblastic differentiation‐related transcription factors in MC3T3‐E1 cells. The shDRG2‐OM group had significantly higher levels in the OSX, RUNX2, SATB2, and DLX5. GM, growth medium; OM, osteoinductive medium. *p < 0.05, **p < 0.01, ***p < 0.001, ****p < 0.0001 versus control; ##*p* < 0.01, ###*p* < 0.001 ####*p* < 0.0001 versus DRG2 knockdown group.*p < 0.05, **p < 0.01, ***p < 0.001, ****p < 0.0001 versus control; ##*p* < 0.01, ###*p* < 0.001 ####*p* < 0.0001 versus DRG2 knockdown group.

Among candidate transcription factors, the expression levels of osterix (OSX), Runt‐related transcription factor 2 (RUNX2), Special AT‐rich sequence‐binding protein 2 (SATB2), and distal‐less homeobox 5 (DLX5) were higher in the shRNA‐OM group than in the control‐OM group. The expression level of OSX was significantly higher in the shRNA‐OM group than the control‐OM group on days 7 and 14 (day 3, *p* < 0.0001; day 7, *p* < 0.0001; day 14, *p* < 0.05). The expression level of OSX was significantly higher in the shDRG2‐GM than in the control‐GM on days 3 and 7 (day 3, *p* < 0.0001; day 7, *p* < 0.001). The expression level of RUNX2 was higher in the shRNA‐OM group than in the control‐OM on day 7 (*p* < 0.0001). The expression level of Runx2 was significantly higher in the shDRG2‐GM group than in the control‐GM group (*p* < 0.01). The expression level of SATB2 was significantly higher in the shDRG2‐OM group than in the control‐OM group on days 3, 7, and 14 (day 3, *p* < 0.05; day 7, *p* < 0.0001; day 14, *p* < 0.05). The expression level of SATB2 was significantly higher in the shDRG2‐GM group than in the control‐GM group on day 7 (*p* < 0.01). The expression levels of DLX5 were significantly higher in the shRNA‐OM group than in the control‐OM group (day 3, *p* < 0.0001; day 7, *p* < 0.0001; day 14, *p* < 0.05). The expression levels of DLX5 were significantly higher in the shDRG2‐GM than in the control‐GM group on days 3 and 7 (day 3, *p* < 0.0001; day 7, *p* < 0.01). The expression level of Activating transcription factor 4 (ATF4) showed no significant difference between the groups (Figure 3).

### 3.2 DRG2 Regulated Osteoblastic Differentiation via Both Canonical and Non‐Canonical Smad Signalling Pathways

Smad1 expression was significantly higher in both the shRNA‐OM and shDRG2‐GM groups compared to their respective controls on day 7 (*p* < 0.001). Similarly, Smad5 levels were significantly elevated in the shRNA‐OM and shDRG2‐GM groups versus controls on day 7 (*p* < 0.01).

**FIGURE 4:**
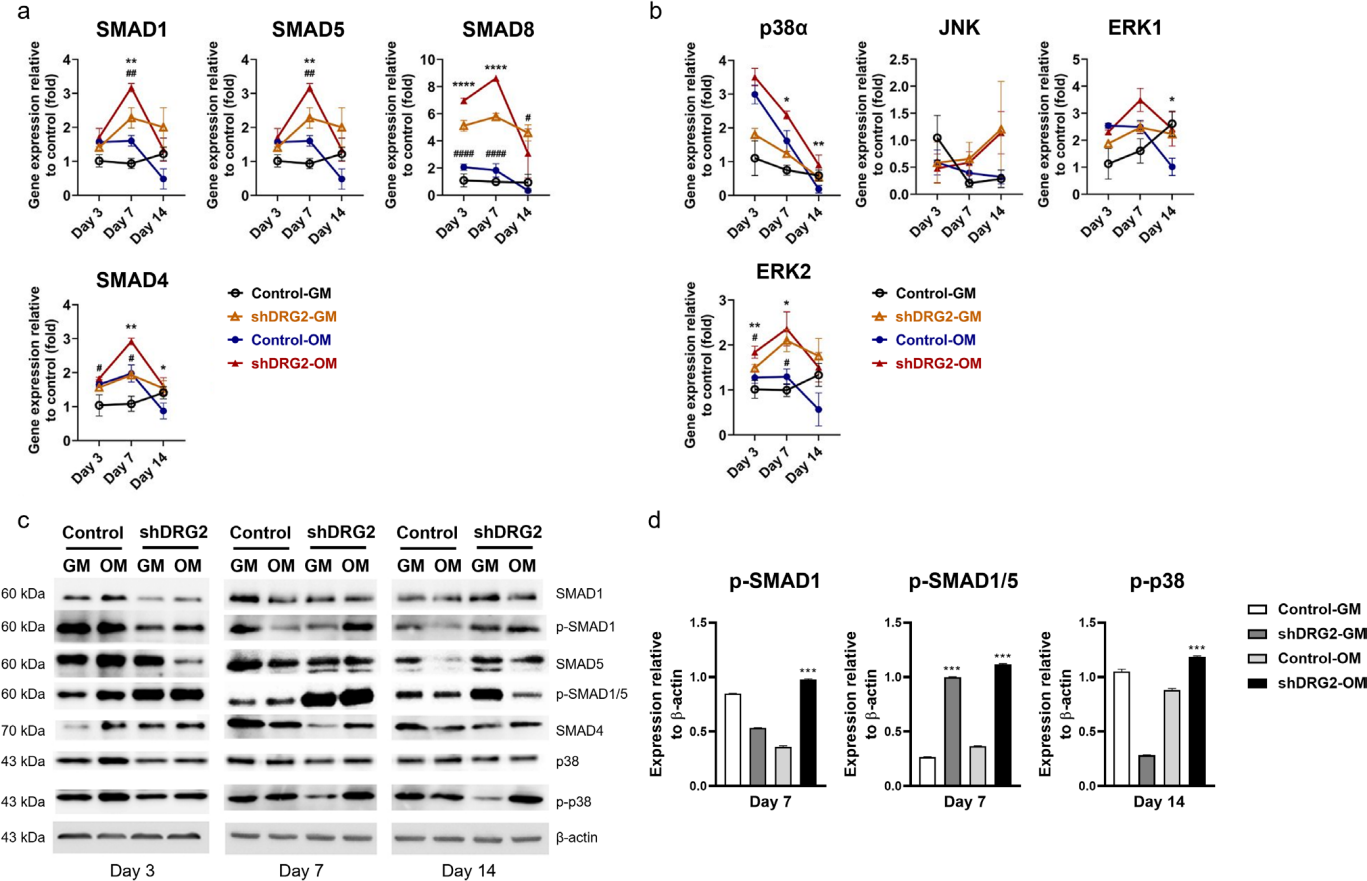
DRG2 regulates osteoblastic differentiation via canonical and non‐canonical BMP signaling at transcript and protein levels. (a) mRNA expression levels of Smad1/4/5/6/8. (b) mRNA expression levels of p38α, JNK, and ERK1/2. (c) Representative Western blot images of phosphorylated Smad1/5/8 (p‐Smad1/5/8) and p38 proteins in shDRG2 and control cells, with β‐actin as loading control. (d) Quantitative densitometry of Western blot bands, showing increased protein levels of p‐Smad1/5/8 and p38 in shDRG2 compared with control. *p < 0.05, **p < 0.01, ***p < 0.001, ****p < 0.0001 versus control; ##*p* < 0.01, ###*p* < 0.001 ####*p* < 0.0001 versus DRG2 knockdown group.

The expression level of common Smad4 was significantly higher in the shDRG2‐OM group than the control‐OM group on day 7 and day 14 (day 7, *p* < 0.01; day 14, *p* < 0.05) and was significantly higher in the shDRG2‐GM than the control‐GM on day 3 and day 7 (*p* < 0.05). The expression level of Smad6 was significantly higher in the shDRG2‐OM than the control‐OM on day 3 and day 7 (day 3, *p* < 0.0001; day 7, *p* < 0.001) and was significantly higher on day 3 and day 7 (*p* < 0.05) (Figure 4a).

The expression level of p38‐alpha was significantly higher in the shDRG2‐OM than the control‐OM on days 7 and 14 (*p* < 0.01). The expression level of ERK1 was significantly higher in the shDRG2‐OM than in the Control‐OM group on day 14 (*p* < 0.05). The expression levels of ERK2 were significantly higher in the shRNA‐OM group than the control‐OM group on days 3 and 7 (*p* < 0.05), and its expression was significantly higher in the shDRG2‐GM than the control‐GM on days 3 and 7 (*p* < 0.05) (Figure 4b). In addition to the transcript data, Western blot analysis confirmed increased phosphorylation of Smad1 and p38 in shDRG2 cells compared with controls (Figure 4c,d). Quantitative densitometry further supported these findings, showing significantly elevated p‐Smad1/β‐actin and p‐p38/β‐actin ratios (Figure 4d). These results provide protein‐level validation that DRG2 modulates both canonical (Smad‐dependent) and non‐canonical (p38‐mediated) BMP signalling pathways.

### 3.3 DRG2 KO Mice Showed Higher Bone Turnover Markers Than the WT Mice

**FIGURE 5:**
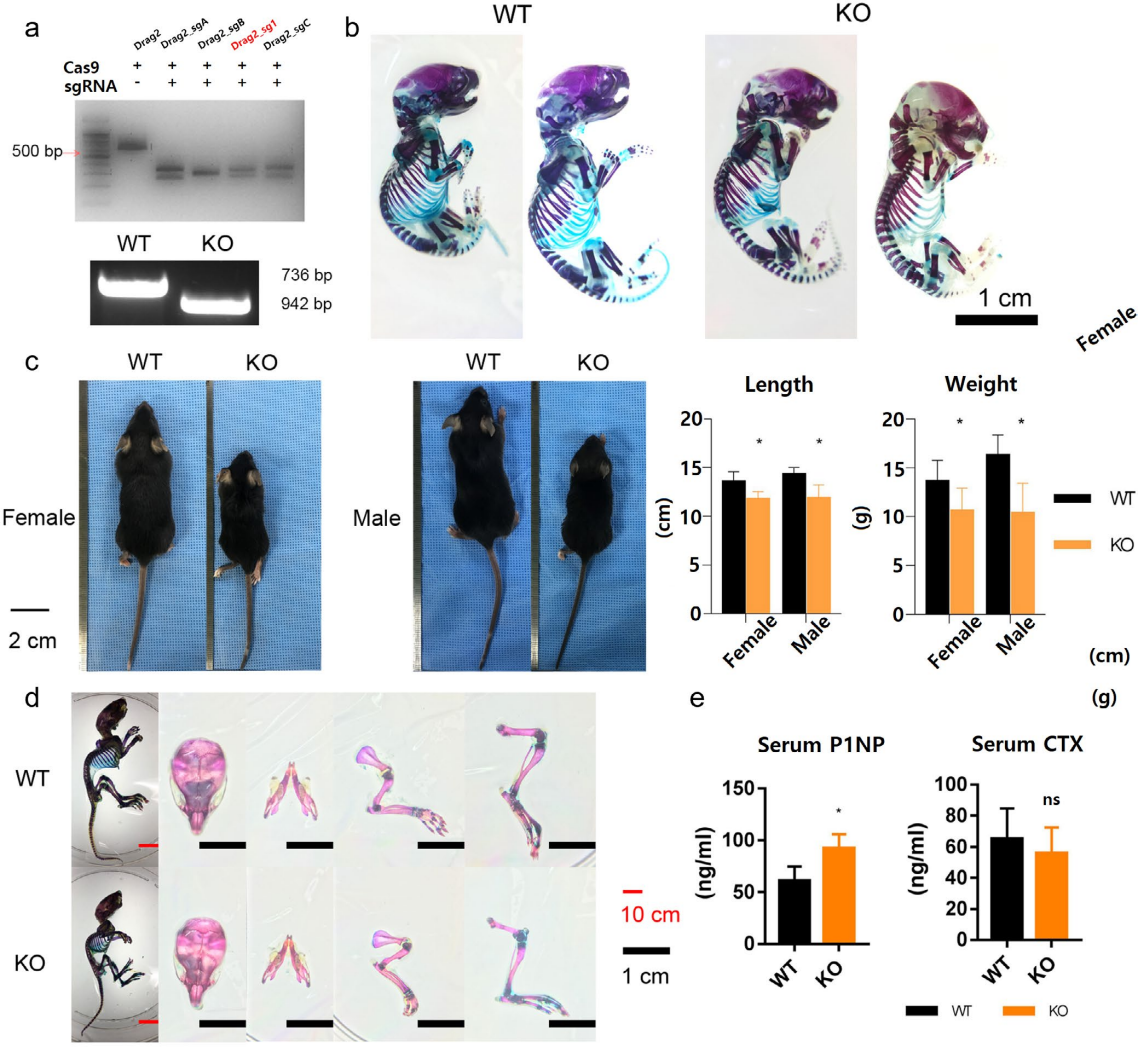
Characterisation of skeletal phenotypes and bone turnover markers in DRG2 knockout and wild‐type mice. (a) Knocking out DRD2 with sgRNA and genomic typing of wild type (WT) and knockout (KO) mice. (b) Alizarin red and alcian blue staining of WT and KO newborn on their postnatal day 1(P1). (c) Length and weight of KO and WT mice on postnatal day 28(P28). *n* = 12 per genotype and sex. (d) Alizarin red and alcian blue staining of WT and KO mouse on their P28. (e) Serum level of procollagen type 1 N‐terminal propeptide (P1NP) and C‐terminal telopeptides type 1 collagen (CTX). *n* = 6. *p < 0.05. ns = not significant.

Following the confirmation of the fabrication of DRG2 knockout (KO) mice (Figure 5a,b). KO mice appear smaller in length and weight (Figure 5c,d). The serum P1NP was significantly higher in the KO group than that in WT (*p* < 0.01), and the level of CTX was lower in the KO group without statistical significance (Figure 5e).

### 3.4 Knocking out of DRG2 Elevates Osteoblastogenesis in Bone Marrow‐Derived MSCs

**FIGURE 6:**
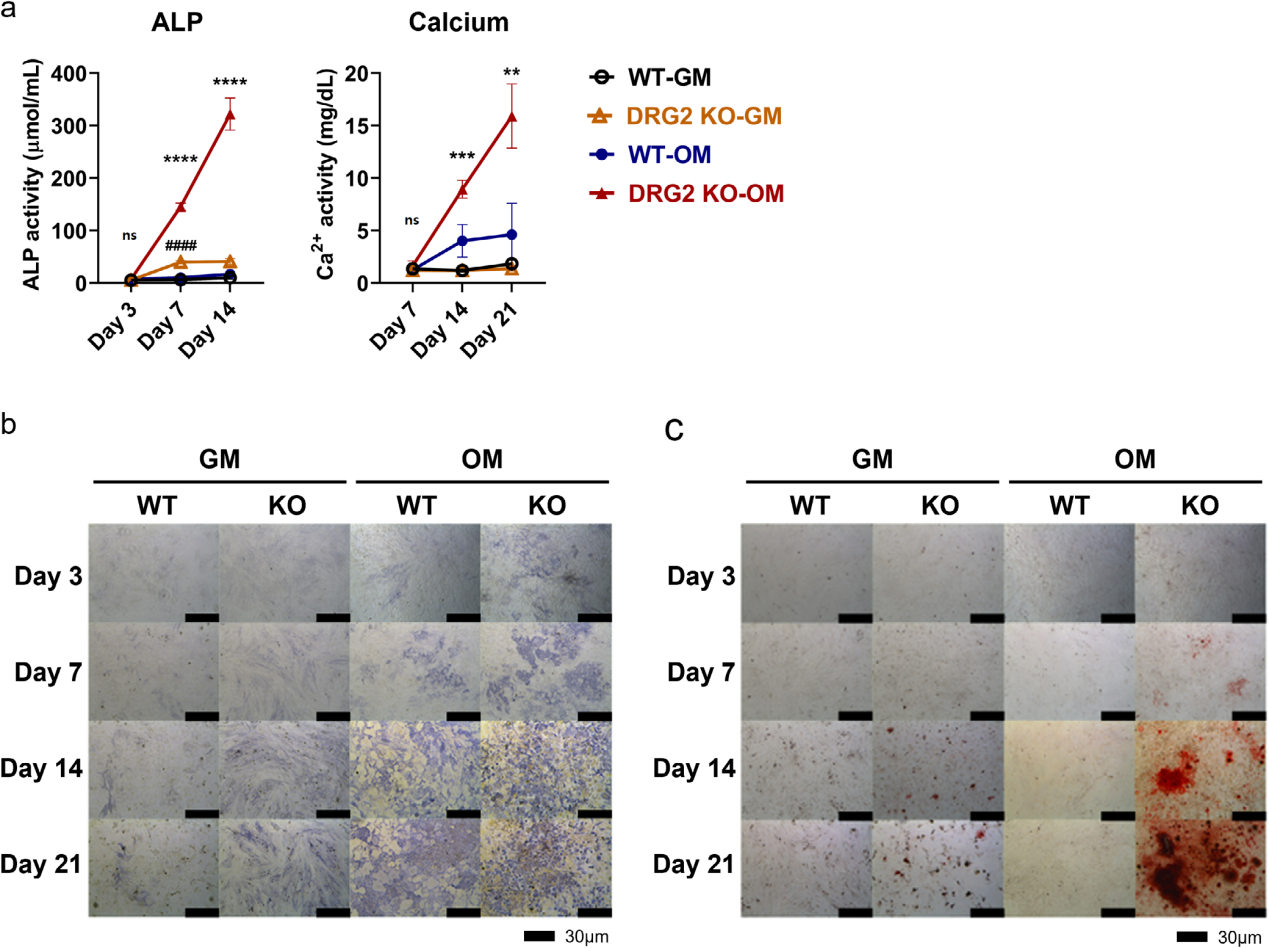
Results of ALP, Calcium assay and staining with respect to DRG2 KO and medium. (a) Result of ALP and Ca assay. The DRG2 KO‐OM group had significantly higher level than the WT‐OM group. (b) The result of ALP staining in BM‐MSCs. The KO‐OM group showed denser staining from day 3. (c) The result of Ca staining in BM‐MSCs. The KO‐OM group showed staining from day 7 after treatment of OM, and the staining were denser than the other groups on all time points. *p < 0.05, **p < 0.01, ***p < 0.001, ****p < 0.0001 versus control; ####*p* < 0.0001 versus DRG2 knockdown group. ns = not significant.

In the ALP assay, the DRG2 KO‐OM group showed significantly higher ALP activity than the WT‐OM group on days 7 and 14 (*p* < 0.0001). The DRG2 KO‐GM group showed significantly higher ALP activity than the WT‐GM group on day 7 (*p* < 0.0001). In the calcium assay, the DRG2 KO‐OM group showed significantly higher calcium levels than the WT‐OM group on days 14 and 21 (*p* < 0.01) (Figure 6a).

After treatment of OM, the MSCs in the KO group showed denser FU staining than that in the WT group from day 3 (Figure 6b). The MSCs in the KO group began to show mineral deposition on day 7 after induction with osteoinductive medium (OM), and on day 21, the mineral deposition almost filled the field of vision. While the WT‐MSCs did not show evident mineralization until day 14, the deposited area and density did not match those of the KO group (Figure 6c).

**FIGURE 7:**
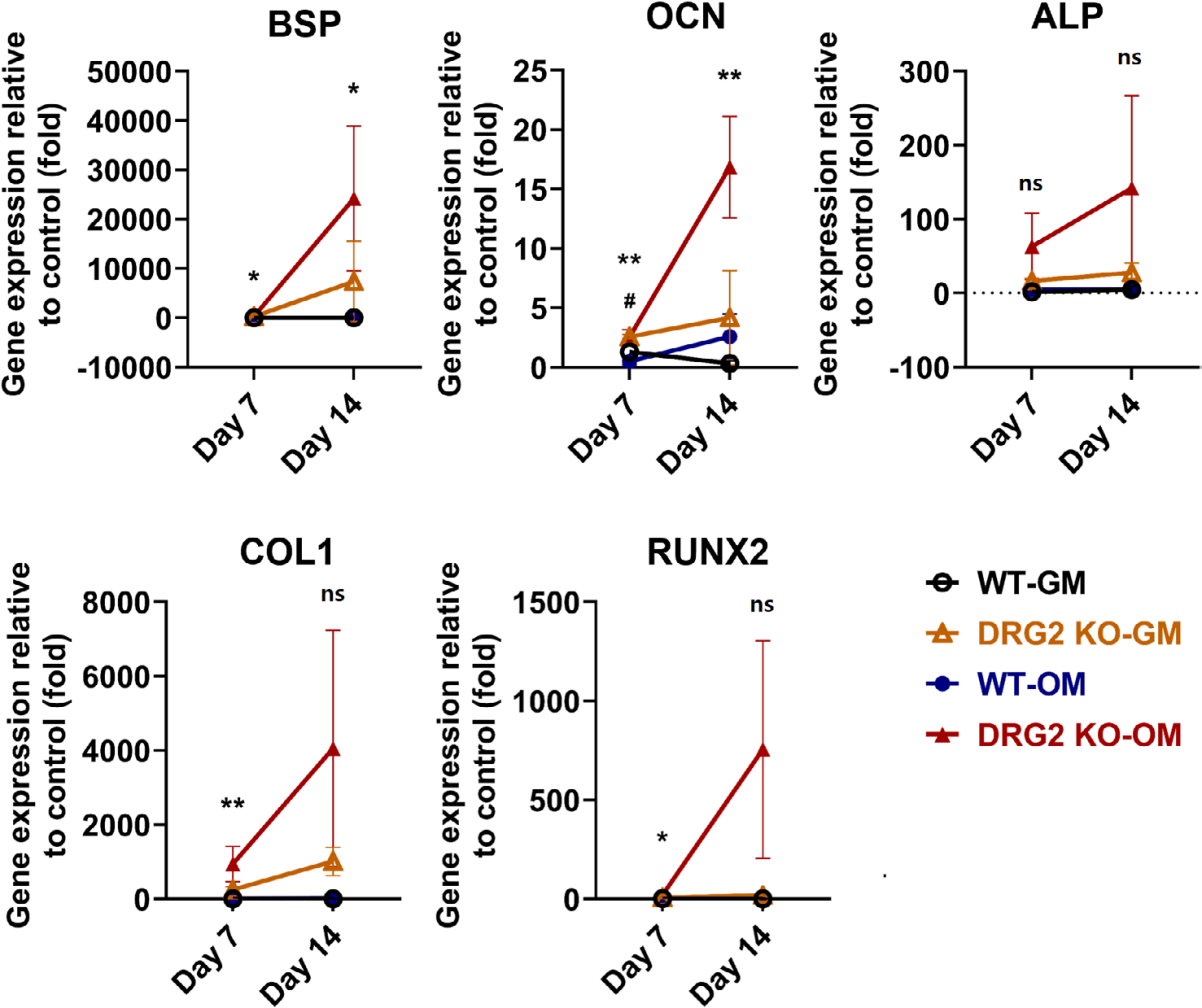
Expression of bone formation markers in BM‐MSCs from DRG2 knockout (KO) and wild‐type (WT) mice. mRNA expression levels of BSP, OCN, and RUNX2 were measured in BM‐MSCs cultured under osteogenic medium (OM) conditions. The DRG2 KO‐OM group showed significantly higher expression of these bone formation markers compared to the WT‐OM and GM groups, particularly on day 7. *p < 0.05, **p < 0.01, versus control; #*p* < 0.05 versus DRG2 knockdown group. ns = not significant.

The real‐time PCR results showed that the DRG2 KO‐OM showed a significantly higher expression level of BSP than the WT‐OM group on days 7 and 14 (day 7, *p* < 0.05; day 14, *p* < 0.05). The expression level of OCN was significantly higher in the DRG2 KO‐OM group than the WT‐OM group on days 7 and 14 (day 7, *p* < 0.01; day 14, *p* < 0.01), and it was significantly higher in the DRG2 KO‐GM than the WT‐GM on day 7 (*p* < 0.05). The expression level of Col1 was significantly higher in the DRG2 KO‐OM group on day 7 (*p* < 0.01). The expression of Runx2 was significantly higher in the DRG2 KO‐OM group than the WT‐OM group on day 7 (*p* < 0.05) (Figure 7).

### 3.5 Knocking out of DRG2 Increased Bone Microarchitecture and BMD in Mice

**FIGURE 8:**
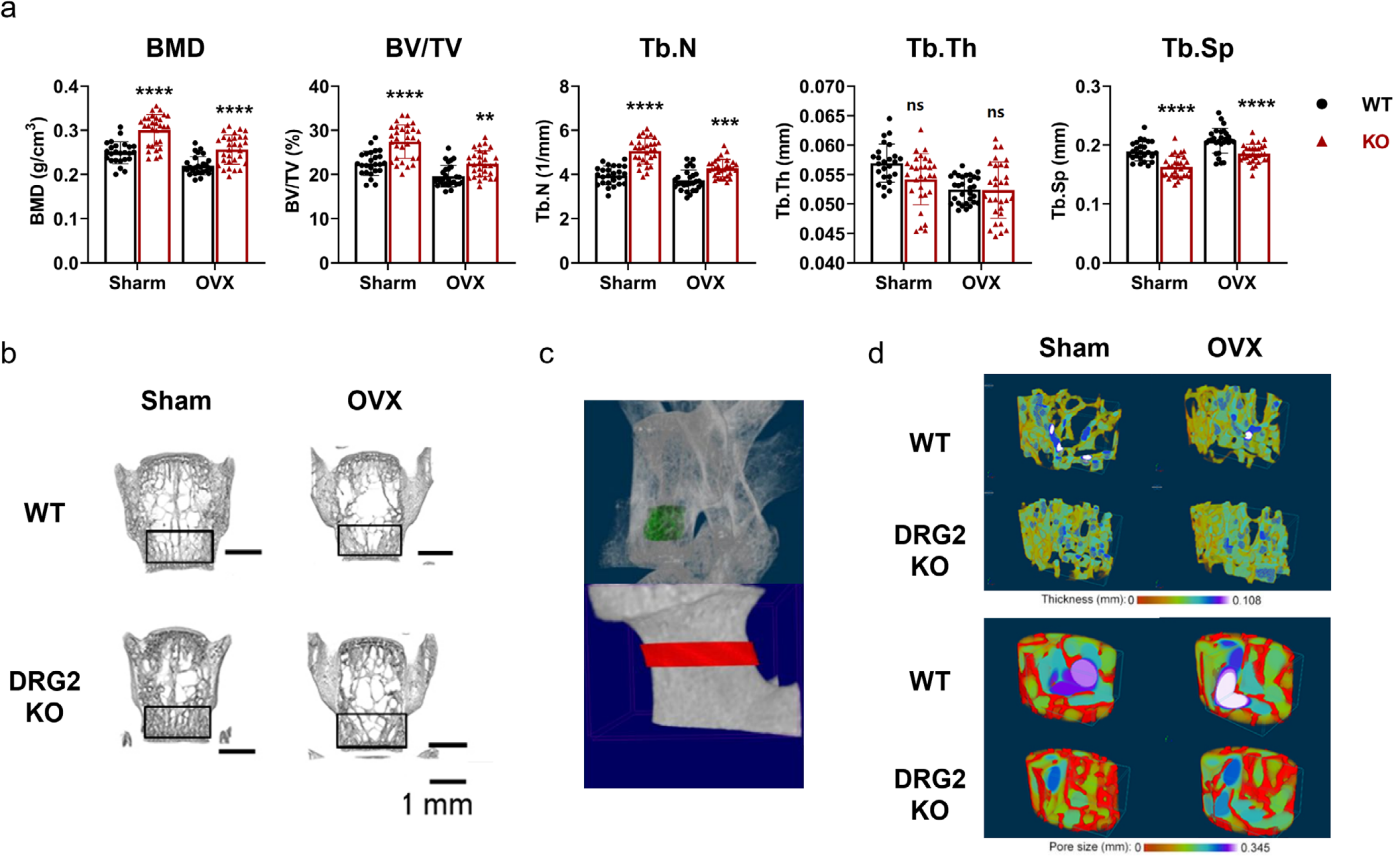
Bone mineral density (BMD) and vertebral trabecular microarchitecture in DRG2 knockout (KO) and wild‐type (WT) mice. BMD was significantly higher in KO mice compared with WT mice under both physiological (sham) and oestrogen‐deficient (OVX) conditions. Group sizes were as follows: Sham‐WT (*n* = 25), sham‐KO (*n* = 30), OVX‐WT (*n* = 28), and OVX‐KO (*n* = 30). (a) Micro‐CT analysis showed that KO mice had significantly higher BV/TV, more Tb.N, and narrower Tb.Sp compared with WT mice. (b) Representative 3D reconstructed images of vertebrae. (c) Region of interest (ROI) used for micro‐CT analysis. (d) Representative images illustrating trabecular bone thickness and separation. *p < 0.05, **p < 0.01, ***p < 0.001, ****p < 0.0001 versus control; ns = not significant.

In the comparison between WT‐sham and KO‐sham, the KO‐sham mice had significantly higher BMD (*p* < 0.0001), BV/TV (*p* < 0.0001), more Tb.N (*p* < 0.0001), narrower Tb.Sp (*p* < 0.0001), and lower trabecular bone thickness (*p* = 0.011) than the WT‐sham mice did. In the comparison of WT‐OVX and KO‐OVX, the KO‐OVX group had significantly higher BMD (*p* < 0.0001), BV/TV (*p* < 0.01), more Tb.N (*p* < 0.001), and narrower Tb.Sp (*p* < 0.0001) than the WT‐OVX group (Figure 8a).

From the reconstructed 3D images of the vertebra, greater trabecular continuity was observed in the KO mice, at the center of the vertebra, and a denser structure at both margins of the vertebra (Figure 8b). The HE staining showed the same trend as the micro‐CT result. The KO mice had a greater number of trabeculae and thinner trabeculae, and narrower separation in both sham and OVX animals (Figure 8d). The separation between the trabecula and trabecula thickness was marked with the spectrum (Figure 8d). As the representative images presented, the WT‐OVX had the brightest sphere between the trabecula, and the KO‐sham and KO‐OVX groups had smaller separations than their counterparts. Instead, the WT groups showed thicker trabeculae than the KO groups in both sham and OVX groups.

**FIGURE 9:**
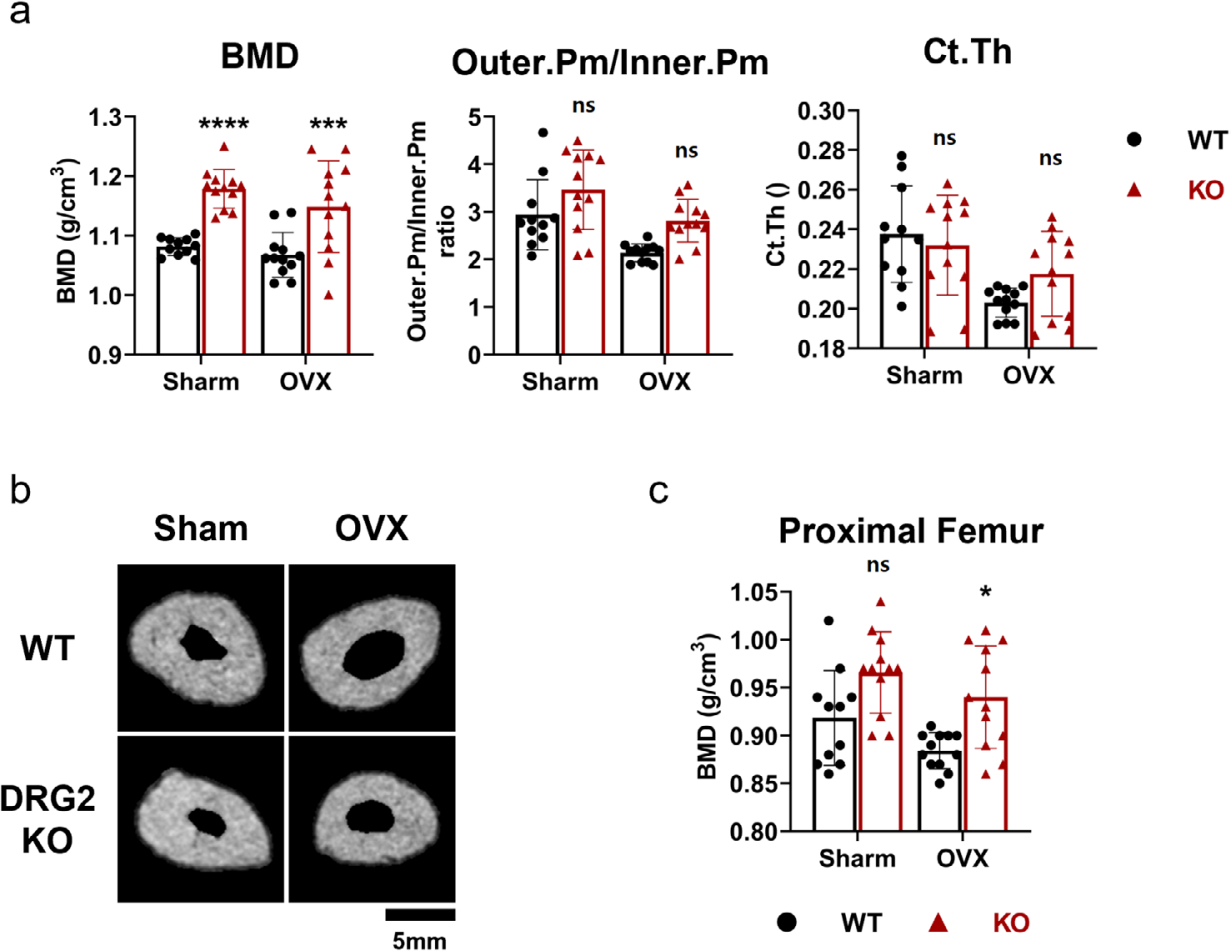
Bone mineral density (BMD) and femoral trabecular microarchitecture in DRG2 knockout (KO) and wild‐type (WT) mice. (a) The BMD was significantly higher in the KO mice than the WT mice under both physiological and ovariectomized conditions (Number of groups: Sham‐WT, 11; sham‐KO, 12; OVX‐WT, 12; OVX‐KO, 12). (b) Representative cross‐sectional images of femur neck. (c) The BMD of KO mice was significantly higher than the WT mice under ovariectomized condition. *p < 0.05, **p < 0.01, ***p < 0.001, ****p < 0.0001 versus control; ns = not significant.

The BMD was significantly higher in the KO‐sham group than the WT‐sham group (*p* < 0.0001) and was significantly higher in the KO‐OVX group than the WT‐OVX group (*p* < 0.001). No significant difference was found in the inner.Pm/outer.Pm ratio or C.Th (Figure 9a). As the representative cross‐sectional images showed, the KO‐sham group had a smaller size, a narrower femur neck cavity, but a denser structure than the WT‐sham group. In the WT‐OVX and KO‐OVX groups, a dilatated cavity was observed in both groups, which was consistent with the micro‐CT analysis (Figure 9b).

The BMD of the proximal femur was significantly higher in the KO‐OVX group than in the WT‐OVX group (*p* < 0.05) (Figure 9c).

## 4 Discussion

Our findings suggest that DRG2 may influence osteoblast differentiation through modulation of key signalling pathways, rather than directly regulating bone metabolism itself. The upregulation of ALP activity, calcium deposition, and osteogenic transcription factors in DRG2‐deficient cells indicates that DRG2 negatively modulates osteoblastogenesis, potentially through BMP, Smad, and MAPK pathways.

When treated with OM, DRG2 knockdown significantly accelerated mineralization by day 14, whereas the control cells exhibited only minimal mineralization by day 21. The expression levels of bone formation‐related genes, such as Col1, Alp, and BSP [20, 21], were significantly elevated in the shDRG2 group. Similarly, transcription factors associated with osteogenesis—including Runx2, OSX, SATB2, and DLX5—were also upregulated. Runx2 and OSX are well‐known markers of early‐stage osteoblasts, while BSP is associated with the transition from preosteoblasts to osteoblasts and bone formation [22]. These data suggest that DRG2 downregulation activates osteoblast differentiation from the early stages of mineralization. Although OCN is expressed at modest levels during the early phases of mineralization, its principal role lies in facilitating the progression of the mineralization process [23] Thus, it can be inferred that the attenuation of DRG2 expression augments mineralization progression via enhanced OCN expression. To explore the upstream signalling responsible for these transcriptional changes, we investigated the role of BMP pathways.

Given BMP's central role in osteoblast differentiation, we evaluated both canonical (Smad‐dependent) and non‐canonical (p38/MAPK‐mediated) pathways [23, 24]. Both signalling pathways converge on transcription factors such as Runx2, and our data indicate increased activity of Smad1/5/8, Smad4, p38α, JNK, and ERK2 in the shDRG2 group. These findings support the hypothesis that DRG2 regulates osteogenesis through modulation of both arms of BMP signalling. In addition to the mRNA findings, we performed protein‐level validation and confirmed increased phosphorylation of Smad1 and p38 by Western blotting, which further supports the involvement of BMP signalling. Although MAPK proteins such as ERK and JNK could not be validated at this stage, their investigation remains an important subject for future studies.

To assess the in vivo relevance of these findings, we fabricated the DRG2 KO mice model. Interestingly, BMSCs derived from DRG2 KO mice exhibited noticeably denser ALP staining compared to the WT group, even in the absence of OM treatment, as early as day 3. Mineralization in the KO group also became evident by day 7 and was markedly greater than in the WT group by day 21. Micro‐CT analysis revealed that DRG2 KO mice had significantly higher BV/TV, Tb.N, and BMD, along with reduced Tb.Sp in the vertebrae and femur. These features suggest enhanced bone formation and improved bone quality in the absence of DRG2. Since bone microarchitecture, BMD, and bone turnover markers are critical predictive factors for osteoporosis and fracture risk [25] DRG2 may influence the development or progression of osteoporosis. Interestingly, trabecular thickness in KO vertebrae was lower than that of wild‐type controls, which we attribute to the smaller overall body size and bone dimensions of KO mice. However, this difference was not observed in ovariectomized mice, and femoral trabecular thickness was higher in the KO group. These findings highlight the need for cautious interpretation, considering systemic growth differences and site‐specific effects.

While the use of a global DRG2 KO model allowed us to evaluate systemic skeletal changes, it also raises the possibility that differences in body size and growth may have contributed to the observed bone phenotypes. Thus, these findings should be interpreted with caution. To clarify tissue‐specific effects, future research should use osteoblast‐specific or conditional DRG2 knockouts. While our findings indicate DRG2 regulates osteogenesis, the lack of rescue experiments means causality cannot be confirmed. We acknowledge this limitation and suggest rescue approaches as a key focus for upcoming studies.

In this study, knockout of DRG2 was found to be related to elevation of the bone microarchitecture and BMD, which are consistent with the previous study that shows overexpression of DRG2 increases survival and cytoskeleton organising of osteoclasts, promotes bone resorption, and may contribute to bone fragility [18].

Moreover, given DRG2's established functions in immune response and mitochondrial dynamics, its involvement in bone remodelling may involve broader systemic or metabolic interactions beyond the canonical osteoblast pathways. These findings may inform future therapeutic strategies targeting DRG2 in osteo‐related disorders.

The limitations of this study include the use of a global knockout mouse model. While effective in simulating the phenotype of patients with DRG2 mutations, this approach does not isolate the specific effects of DRG2 deletion in bone tissue. In addition, although we validated DRG2's influence on canonical and non‐canonical BMP pathways at both transcript and protein levels, the exact molecular mechanisms by which DRG2 interacts with these pathways remain unclear. Further investigation is needed to clarify whether DRG2 directly modulates BMP signalling components or acts indirectly through other regulators.

## 5 Conclusion

This study provides strong evidence that DRG2 plays a role in osteoblast differentiation and bone microarchitecture; however, further mechanistic studies are needed to determine whether this is a direct effect or mediated through other regulatory pathways. Given the complexity of DRG2's functions, its influence on bone metabolism should be interpreted within the signalling pathways involved in bone formation and resorption.

## Author Contributions

**Yuan‐Zhe Jin:** formal analysis (equal), investigation (equal), methodology (equal), visualization (equal), writing – original draft (equal), writing – review and editing (equal). **Minjoon Cho:** investigation (equal), methodology (equal), supervision (equal), validation (equal), writing – original draft (equal), writing – review and editing (equal). **Jae Hyup Lee:** conceptualization (equal), data curation (equal), funding acquisition (equal), methodology (equal), project administration (equal), resources (equal), supervision (equal), validation (equal), writing – review and editing (equal).

## Ethics Statement

All procedures involving the use of animals were officially approved.

## Consent

Written informed consent for publication was obtained from all participants.

## Conflicts of Interest

The authors declare no conflicts of interest.

## Supporting information


**Figure S1:** Schematic diagram of experiment design of transgenic mice.
**Table S1:** Primers used in this study.

## Data Availability

The data that support the findings of this study are available from the corresponding author on reasonable request.
