# Supplementary material for: Effect of Depletion of Developmentally Regulated GTP Binding Protein on Osteoblastic Differentiation and Bone Microarchitecture
Source: J Cell Mol Med. 2025 Oct 29;29(21):e70895. doi: 10.1111/jcmm.70895 (PMC12571191; doi:10.1111/jcmm.70895)
Supplement: Supplementary file 1 — Figure S1: Schematic diagram of experiment design of transgenic mice. Table S1: Primers used in this study. [file JCMM-29-e70895-s001.docx]

# Supplementary Materials:

**Detailed protocols of *in vitro* study**

# 1. Culturing and shRNA transfection of MC3T3-E1 cell

To investigate the function of DRG2 in osteoblastic differentiation, we diminished the expression of DRG2 in mouse pre-osteoblast MC3T3-E1 cells with shRNA. The shRNA (SANTA CRUZ BIOTECHNOLOGY, INC) was used following the manufacturer's instruction. For transfection, Lipofectamine (Thermo Fisher Scientific) containing 1 μg DRG2 shRNA Plasmid was mixed into opti-MEM medium, and the mixture was incubated at room temperature for 30 minutes.

The shRNA plasmid transfection medium was added to each well in a 6-well plate. Then the cells were incubated in the standard environment (5% CO2, 37 ºC) for 5 hours. For selecting stably transfected cells, 10 μg/ml puromycin was added into the basic culture medium, i.e., alpha-MEM containing 10% FBS and 1% antibiotic antimycotic (Thermo Fisher Scientific). The medium was changed every 2-3 days.

# 2. Inducing osteoblastic differentiation in MC3T3-E1 cells

MC3T3-E1 cells were induced with α-MEM (containing 10% FBS and 1% antibiotic antimycotic) based osteoinduction medium that contained 10^-8^ M dexamethasone, 100 μmol ascorbic acid, and 10 mM β-glycerophosphate.

# 3. ALP staining and ALP activity assay

For ALP staining, fast blue RR salt was dissolved in distilled water, and Naphthol AS-MX phosphate alkaline solution was diluted in the water in a ratio of 1:24. Cells were washed twice with DPBS. The fast blue RR salt/Naphthol AS-MX phosphate alkaline solution was then added, followed by incubation at room temperature for 5 minutes. Staining was observed under optical microscopy (Leica).

ALP activity was measured with a spectrophotometer. Cells were washed twice with DPBS and lysed with 0.2% Triton X-100. Cell lysates were assayed for ALP activity using p-nitrophenylphosphate as a substrate. ALP activity was defined as the amount of p-nitrophenol released after incubation for 30 min at room temperature. The color change was measured spectrophotometrically at 405 nm, and the experiment was biologically repeated 3 times.

# 4. Calcium staining and assay

Wells were washed twice with DPBS, added with 40nM alizarin red solution, and incubated at room temperature for 5 minutes. The mineral deposition was observed with optical microscopy (Leica).

Cells were lysed with 0.2 ml 0.5% HCl and 2.5 μl of the sample was transferred into a 96-well plate. An equal volume of reagent A and reagent B from QuantiChrom calcium assay kit (BioAssaySystems, Hayward, CA, USA) was mixed, and 200 μL of the mixture was added into each well. After incubating at room temperature for 3 minutes, the optical density at 612 nm was measured with an ELISA reader. The experiment was biologically repeated 3 times.

# 5. Real-time PCR

RNAs were extracted using Qiagen RNeasy mini prep kit(Life Technology) and reverse transcribed into cDNA using Maxime™ RT PreMix kit (Intron technology). Real-time PCR was performed with SYBR Green and LightCycler® 480 Instrument (Roche Life Science) real-time PCR system. The expression level was calculated as -2^ΔΔCt^, and the experiment was biologically repeated 3 times. The expression was corrected using GAPDH. ^(12)^ Primers used are listed in Supplementary Table S1.

# 6. Semiquantitative RT-PCR

The expression level of DRG2 was monitored with semi-quantitative RT-PCR, with the primer kit from SANTA CRUZ BIOTECHNOLOGY, INC (product number DRG2 (m):sc-143171-PR).

Figure S1. Schematic diagram of experiment design of transgenic mice.


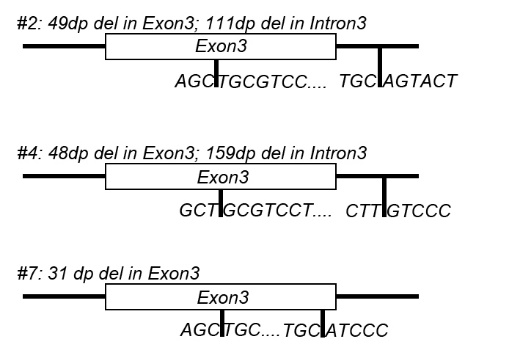


Table. S1. Primers used in this study.

| mouse Drg2 | F-AGGCAGGTCCCTTACCTGAG |
| --- | --- |
|  | R-AGATGTGCAGAGGGCAAAGA |
| human Drg2 | F-ACATTCTTGAGTCTGATGACCT |
|  | R-AGTAGATGTTAGGCTTGTGCTT |
| Dlx5 | F-CCGCTTTACAGAGAAGGTTTCA |
|  | R-TCTTCTTGATCTTGGATCTTTTGTT |
| Smad1 | F-TGAAAACACCAGGCGACATA |
|  | R-TGAGGCATTCCGCATACAC |
| Smad5 | F-GCAGTAACATGATTCCTCAGACC |
|  | R-GCGACAGGCTGAACATCTC |
| Smad8 | R-CGGATGAGCTTTGTGAAGG |
|  | F-GGGTGCTCGTGACATCCT |
| Smad4 | R-AAGCTGCCCTGTTGTGACTGT |
|  | F-GGAGAGTTGACCCAAGCAAAAG |
| Smad6 | F-GTTGCAACCCCTACCACTTC |
|  | R-GGAGGAGACAGCCGAGAATA |
| OPN | F-GATGATGATGACGATGGAGACC |
|  | R-CGACTGTAGGGACGATTGGAG |
| Collagen I | F-ATCTCCTGGTGCTGATGGAC |
|  | R-ACCTTGTTTGCCAGGTTCAC |
| BSP | F-GAGACGGCGATAGTTCC |
|  | R-AGTGCCGCTAACTCAA |
| p38 | F-CCCAGCAACCTAGCTGTG |
|  | R-GCTCGGTACCACCTGGTAG |
| JNK | F-TCCCAGCTGACTCAGAGCAT |
|  | R-GCTTCATCTACGGAGATCCTT |
| ERK1 | F-CCTGCTGGACCGGATGTTA |
|  | R-TGAGCCAGCGCTTCCTCTAC |
| ERK2 | F-GGAGCAGTATTATGACCCAAGTGA |
|  | R-TCGTCCACTCCATGTCAAACT |
| Gapdh | F- ACCCAGAAGACTGTGGATGG |
|  | R- CACATTGGGGGTAGGAACAC |
